# Investigating difficult to detect pancreatic lesions: Characterization of benign pancreatic islet cell tumors using multiparametric pancreatic 3-T MRI

**DOI:** 10.1371/journal.pone.0253078

**Published:** 2021-06-11

**Authors:** Kwadwo Antwi, Patricia Wiesner, Elmar M. Merkle, Christoph J. Zech, Daniel T. Boll, Damian Wild, Emanuel Christ, Tobias Heye

**Affiliations:** 1 Department of Radiology, University Hospital Basel, Basel, Switzerland; 2 Center for Neuroendocrine and Endocrine Tumors, University Hospital Basel, Basel, Switzerland; 3 Division of Endocrinology, Diabetology and Metabolism, University Hospital Basel, Basel, Switzerland; Humanitas Clinical and Research Center - IRRCS, ITALY

## Abstract

**Introduction:**

Pancreatic islet-cell tumors (PICT) often present with atypical signal-characteristics and are often missed on preoperative imaging. The aim of this study is to provide a multiparametric PICT characterization and investigate factors impeding PICT detection.

**Material and methods:**

This is a detailed MRI analysis of a prospective, monocenter study, including 49 consecutive patients (37 female, 12 male; median age 50) with symptoms due to endogenous hyperinsulinemic hypoglycemia (EHH) and mostly negative prior-imaging. All patients received a 3-T MRI and a ^68^Ga-DOTA-exendin-4-PET/CT. Pooled accuracy, sensitivity, specificity and inter-reader agreement were calculated. Reference-standard was histopathology and ^68^Ga-DOTA-Exendin-4-PET/CT in one patient who refused surgery. For PICT analyses, 34 patients with 49 PICTs (48 histologically proven; one ^68^Ga-DOTA-exendin-4-PET/CT positive) were assessed. Dynamic contrast-enhanced (DCE) Magnetic Resonance Images (MRI) with Golden-Angle-Radial-Sparse-Parallel (GRASP) reconstruction, enabling imaging at high spatial and temporal resolution, was used to assess enhancement-patterns of PICTs. Tumor-to-background (T2B) ratio for each sequence and the employed quantitative threshold for conspicuity of PICTs were analyzed in regard to prediction of true-positive PICTs.

**Results:**

Evaluation of 49 patients revealed a pooled lesion-based accuracy, sensitivity and specificity of 70.3%, 72.9% and 62.5%, respectively. Mean PICT size was 12.9±5.3mm for detected, 9.0±2.9mm for undetected PICTs (p-value 0.0112). In-phase T1w detected the most PICT (67.3%). Depending on the sequence, PICTs were isointense and poorly visible in 29–68%. Only 2/41(4.9%) PICTs showed typical signal-characteristics across T1w, T2w, DWI and ceT1w combined. 66.6% of PICTs enhanced simultaneously to the parenchyma, 17.8% early and 15.6% late. Predictor screening analysis showed number of sequences detecting a PICT, lesion size and in-phase T1w T2B ratio had the highest contribution for detecting a true-positive PICT.

**Conclusion:**

The majority of PICTs enhance simultaneously to surrounding parenchyma, present with atypical signal-characteristics and thus are poorly visible. In non-enhancing PICTs, radiologists should search for small lesions most likely conspicuous on unenhanced T1w or DWI.

## Introduction

Benign insulinomas are pancreatic islet cell tumors (PICT) of clinical importance as they are the most prevalent cause of endogenous hyperinsulinemic hypoglycemia (EHH) in adult patients [1]. Exact preoperative localization is a prerequisite for minimal invasive surgery in order to improve postoperative outcome [1–4]. The role of imaging in the diagnostic work-up of patients with PICTs is to localize the culprit lesion since the diagnosis is often biochemically established.

A systematic review reported a mean sensitivity in insulinoma detection for arterial calcium stimulation with venous sampling (ASVS), endoscopic ultrasound (EUS), magnetic resonance imaging (MRI) and computed tomography (CT) to be 85%, 76%, 58% and 54% respectively [5]. 3-Tesla (3T) MRI seems to be more accurate in the detection of insulinomas than contrast-enhanced computed tomography (ceCT), as it demonstrates higher tumor conspicuity and depicts tumor-to-duct distance more accurately, making 3T MRI the preferable morphological imaging method [6]. The typical MRI appearance of PICTs in commonly used sequences is described as hyperintense on T2-weighted (T2w) sequences, hypointense on T1-weighted (T1w) sequences, and arterial enhancing on contrast-enhanced (ce) T1-weighted images [7]. However, a considerable proportion of PICTs present with atypical signal characteristics [8,9].

It was previously shown that routine CT or MRI performed by referring centers had a diagnostic accuracy of 40% in comparison to ^68^Ga-DOTA-exendin-4-PET/CT with 93.9% [10]. The low performance of CT/MRI may be due to a selection bias as mainly difficult cases with negative or non-conclusive CT or MRI imaging results were included [10]. This suggests that a subgroup of PICTs is challenging to localize on cross-sectional imaging either due to their small size or atypical signal characteristics.

Therefore, the aim of this study was to provide a multiparametric lesion characterization in patients with difficult to detect PICTs and to investigate factors that impede PICT detection on MR imaging. To our knowledge, this the first study in which the enhancement pattern of PICTs was assessed with DCE-MRI using GRASP reconstruction depicting the entire range of unenhanced, arterial, portal venous and late phase. The hypothesis is that not all PICTs have a typical MR imaging presentation, and a relevant proportion of PICTs show atypical signal- and variable enhancement characteristics, and thus are less likely to be detected.

## Materials and methods

### Study design

This is a detailed MRI analysis of a prospective, monocenter, imaging study. The study was approved by the Regional Scientific Ethics Committee and patients provided written consent in accordance with the Declaration of Helsinki prior to participation. The study was funded by the Swiss National Science Foundation (grant number 320030–152938) and Desirée & Niels Yde’s Foundation (grant number 389–12).

**Fig 1:**
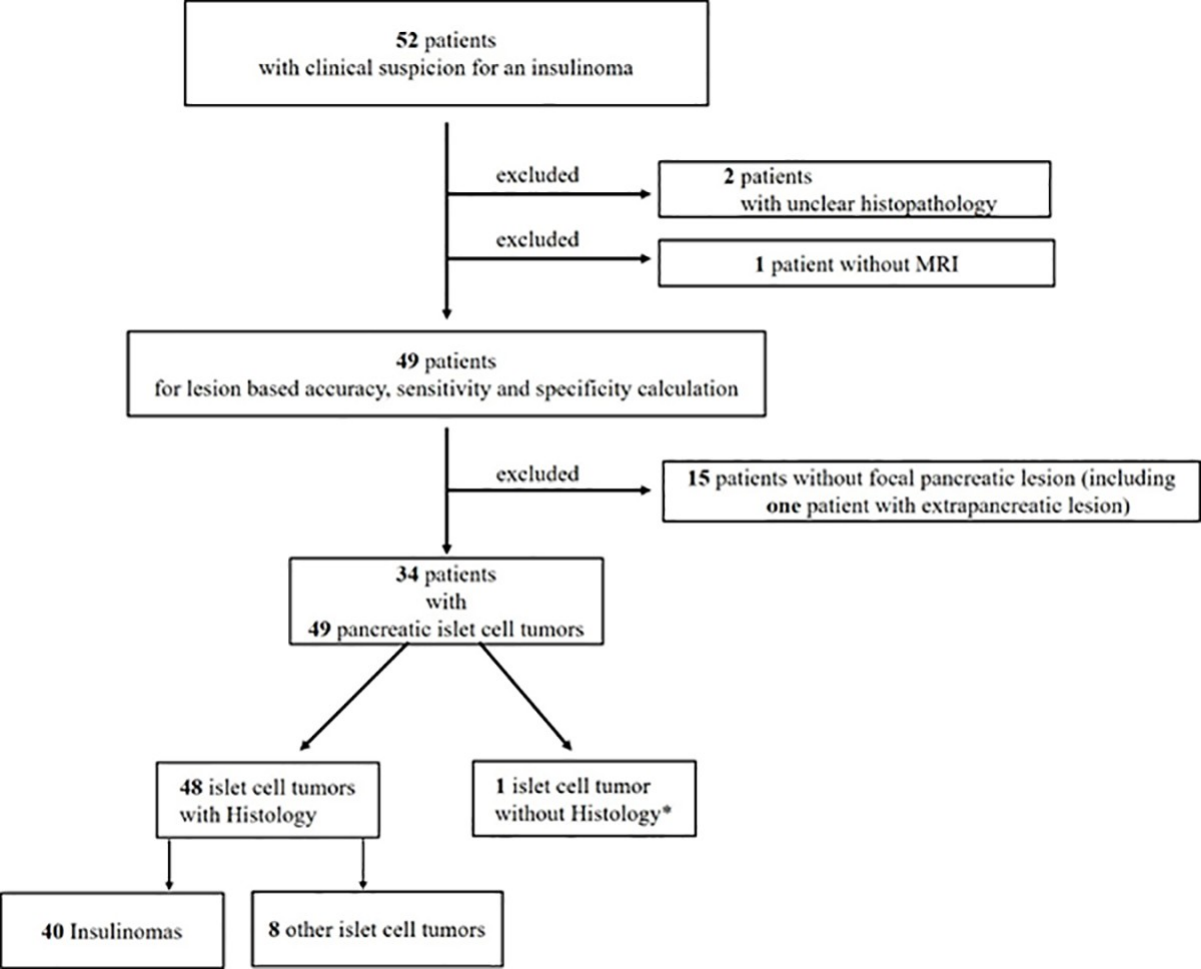
Study sample flow diagram. This is the outline of the selection of the final study sample with inclusion and exclusion criteria within the defined observation window. * ^68^Ga-DOTA-Exendin-4 PET/CT served as reference standard. PICT = pancreatic islet cell tumor.

52 consecutive study patients with EHH highly suspicious for the presence of an insulinoma (Fig 1) were recruited between January 2014 and March 2017 and referred to the University Hospital Basel (ClinicalTrials.gov, NCT02127541).

Inclusion criteria for patients were biochemically proven EHH with neuroglycopenic symptoms. Signs of malignancy (defined as metastases in extrapancreatic organs), renal insufficiency, pregnancy or breastfeeding in women were exclusion criteria.

### Recruitment

Patients were characterized by symptoms related to hypoglycemia prior to enrollment (symptom duration median 24 months, interquartile range 14–57 months, Table 1). Patients received various, mainly negative external imaging procedures prior to enrollment, including MRI in 37/52 patients (71.2%), CT in 24/52 patients (46.2%), endoscopic ultrasound in 33/52 patients (63.5%) including five patients with biopsy, Somatostatin receptor imaging in 18/52 patients (34.6%), ^18^F-DOPA PET/CT in 8/52 patients (15.4%), arterial calcium stimulation with venous sampling in 10/52 patients (19.2%) and surgery with intraoperative ultrasound in 6/52 patients (11.5%). Sensitivity and accuracy of prior external CT/MRI were 38.2% and 40%, respectively (per participant analysis) [10].

**Table 1:** Demographics and characteristics in EHH patients.

Characteristics	
**median age (years); Range**	50 (19–81)
**Sex**	
Men (%)	12/49 (24.5%)
Women (%)	37/49 (75.5%)
**MEN-1**	
yes	6/49 (12.2%)
no	43/49 (87.8%)
**Surgery**	
yes	37/49 (75.5%)
no	12/49 (24.5%)
Duration of symptoms (months; not including 2 controls)	24 (14–57)

This is the demographics and characteristics in 49 patients. Data are median (IQR) or number/total (%).

Fig 1 shows the recruitment process and Table 1 shows the baseline characteristics of the study patients. All 52 patients received a ^68^Ga-DOTA-Exendin-4 PET/CT. One patient could not undergo 3T MRI due to pacemaker implantation and 2 patients had to be excluded due to unclear histopathology. Thus 49 patients were analyzed for lesion-based accuracy, sensitivity and specificity calculation.

In 15 patients, no PICT compatible lesion within the pancreas could be identified (including one patient with a clear extrapancreatic PICT). Six patients had known MEN-1-syndrome, which typically presents with multiple PICTs. Therefore, in total 34 patients with 49 pancreatic islet cell tumors were analyzed.

48 PICT compatible lesions were resected with a histopathological confirmation, 40 were compatible with benign insulinoma, eight were compatible with other PICT (mostly non-functioning neuroendocrine tumors). One patient was classified as having no detectable lesion since histopathology showed nesidioblastosis, a condition represented by abnormal islet cells scattered across the pancreas. Due to refusal to surgery in one study participant, one PICT compatible focal lesions was not resected but was confirmed with the reference standard ^68^Ga-DOTA-Exendin-4-PET/CT.

### Study protocol

All patients underwent a contrast-enhanced 3-T MRI examination using a standardized protocol and a ^68^Ga-DOTA-exendin-4 PET/CT performed within 4 days.

#### 3-T MRI protocol

Abdominal 3-T MRI acquisition was performed on a commercially available 3T system (MAGNETOM Prisma, Siemens Healthineers, Erlangen, Germany) in supine position using a multichannel body surface coil. The body surface coil was placed firmly across the abdomen and patients were asked to breathe calm and shallow to avoid excessive abdominal excursion during breathing. The protocol aimed at a high spatial resolution and robustness with regard to breathing and motion artifacts employing breath-hold and gated free-breathing sequence techniques. Details of the MR imaging protocol are given by Table 2. Unenhanced T1w Dixon VIBE (T1w) sequences were acquired twice (before and after free-breathing diffusion-weighted and T2w imaging) in every participant.

**Table 2:** Multiparametric pancreatic 3-T MRI sequence parameter.

Parameter	T2w HASTE	T2w HASTE	fs T2w TSE	T1w Dixon VIBE	DWI*	fs T2w BLADE	ce GRASP**	ce T1w Dixon VIBE	ce T1w Dixon VIBE
Orientation	coronal	transverse	transverse	transverse	transverse	transverse	transverse	transverse	coronal
breathing	Breath hold	Breath hold	Breath hold	Breath hold	Free breathing	gated	Free breathing	Breath hold	Breath hold
Repetition time/echo time (msec)	1400/92	1600/117	2800/101	3.9/1.28;2.51	6000/58	3287/90	3.48/1.63	4.2/2.1	4.2/1.35
Slice thickness (mm)	4	4	3	2.5	4	4	2.5	2.5	1.5
Intersection gap (mm)	4.8	4.8	3.6	0	4.8	4.8	0	0	0
Matrix	256x256	320x170	320x210	320x170	192x156	320x320	256x256	320x170	320x216
Flip angle (degree)	160	160	134	9	90	155	12	9	9

Note

TSE = Turbo Spin Echo.

HASTE = Half fourier-Acquired Single shot Turbo spin Echo.

BLADE = multi-shot Turbo Spin Echo sequence using periodically rotated overlapping parallel lines with enhanced reconstruction.

VIBE = Volumetric Interpolated Breath-hold Examination.

DWI = diffusion-weighted imaging using five b values (0, 50, 200, 400, and 800 s/mm^2^).

GRASP = golden-angle radial sparse parallel; a sequence which allows a high temporal and spatial resolution with continuous acquisition in shallow free breathing [11].

**Temporal resolution for golden-angle radial sparse parallel was 7 seconds per volume with 55 spokes per frame over 3 minutes and 45 seconds acquisition.

ce = contrast enhanced.

fs = fat saturation.

The Golden-Angle Radial Sparse Parallel (GRASP) sequence using a compressed sensing technique with retrospective breathing excursion gating [11] and a resulting temporal resolution of 7 seconds per volume was used to continuously image over the course of 3 minutes and 45 seconds after dynamic injection of 0.1 mmol Gd-DOTA (Dotarem, Guerbet, Villepinte, France) per kilogram body weight.

#### PET/CT protocol

PET/CT was performed on different scanners in supine position: PET/16-detector CT scanner (Discovery ST; GE Healthcare, Chicago, USA), PET/64-detector CT scanner (Discovery ST; GE Healthcare, Chicago, USA), PET/128-detector CT scanner (Biograph mCT-X RT Pro Edition, Siemens Healthineers, Erlangen, Germany). One bed position of the upper abdomen was acquired during 8 min, 2.5 h after the intravenous injection of ^68^Ga-DOTA-exendin-4. PET images were reconstructed using an ordered-subsets expectation maximization (OSEM) algorithm with three iterations and 25 subsets. Low-dose CT (120 kVp, 30–100 mAs) was used in all patients for attenuation correction and to provide an anatomic reference.

### Qualitative analysis

Three board-certified radiologists (E.M.M., C.J.Z., D.T.B.), each with >10 years of experience in abdominal MRI post-board certification, independently assessed 3T MRI examinations on a commercially available picture archiving and communication workstation (Centricity PACS RA 1000; GE Healthcare, Chicago, USA) in random order. The dynamic GRASP sequences were evaluated on a post-processing solution (Syngo.via, VB20, Siemens Healthineers, Erlangen, Germany). All readers were unaware of the patients’ identity, other imaging results, and the patient’s clinical history.

If a PICT was considered to be present, the location and signal intensity (hypo-, iso- or hyperintense relative to normal pancreatic parenchyma) of each PICT on each sequence was recorded. Typical PICT MRI characteristics are defined as hypointense on T1w sequences, hyperintense on T2w sequences, hyperintense on high b-value DWI sequences, and hypervascular on contrast-enhanced T1w images [7]. In patients having more than one PICT, it was assumed that lesions are biologically independent from each other and can exhibit different signal characteristics.

### Quantitative analysis

Two non-blinded radiology residents with more than three years of experience (K.A.,P.W.) who had knowledge of the precise location of the PICT on the basis of the ^68^Ga-DOTA-Exendin-4 -PET/CT or histology, measured the size and the signal intensity of the PICT and the head, body and tail of the normal pancreas parenchyma using regions of interest (ROI) in a post-processing software (Syngo.via, VB20, Siemens Healthineers, Erlangen, Germany). The tumor-to-background (T2B) ratio was calculated for each sequence, the background being the mean of the intensity of the normal parenchyma in the caput, corpus and cauda.

PICTs with a T2B ratio of < 0.8 were classified as hypointense relative to the normal parenchyma and PICTs with a T2B ratio of > 1.2 as hyperintense. Therefore, these PICTs should be well demarcated and visible. PICTs with a T2B ratio ranging from 0.8–1.2 were defined as isointense relative to normal parenchyma and, therefore, poorly demarcated and visible.

When no PICT compatible lesion was visible on MRI and PET/CT data and the PICT was confirmed by surgery only, no ROI was placed and no measurements were taken. Temporal enhancement was defined as simultaneous enhancement (isotime) between >-1 and <1 seconds of the recorded peak enhancement time point, early enhancement consequently ≤-1 and late as ≥ 1 seconds comparing the time of maximum enhancement of the PICT to the normal pancreas parenchyma. Given the native GRASP temporal resolution of 7 seconds, the temporal enhancement definition of isotime reflects a temporal interval of 7 seconds ± 1 second.

### Reference standard

The reference standard for the qualitative analysis including 49 EHH subjects was histopathological diagnosis and/or ^68^Ga-DOTA-Exendin-4 -PET/CT if an evident focal uptake with a tumor to background ratio > 1.2 SUV (standardized uptake value) was identified. The reference standard for the quantitative lesion analysis including 33 subjects was histopathology. In one patient where surgery was not performed ^68^Ga-DOTA-Exendin-4-PET/CT was used as reference standard.

### Statistical analysis

Statistical analysis was performed using commercially available software (JMP 13.0, SAS, Cary, NC, USA). Descriptive data analysis is displayed as percentages and means with standard deviations. The qualitative and quantitative analyses are both lesion based analyses. To analyze pooled accuracy, sensitivity, specificity, false discover rate and false negative rate of the three readers, the results were derived by combining lesion based results from all readers as a majority decision (at least 2 of 3 readers with the same decision). A two-sided p-value of less than 0.05 was considered to indicate a significant difference. Statistical difference testing was performed using the Wilcoxon signed-rank test. For group comparisons one-way ANOVA was performed and P values less than 0.05 were considered statistically significant.

Simple chance-corrected κ coefficients were used to assess inter-reader agreement for PICT detection and characterization. 0.00–0.20 indicated slight agreement; 0.21–0.40, fair agreement; 0.41–0.60, moderate agreement; 0.61–0.80, substantial agreement; and 0.81–1.00, almost perfect agreement [12]. The impact of the employed quantitative threshold for conspicuity of PICTs was analyzed in regard to the prediction of true positive PICTs. For this, a decision tree analysis was performed, including T2B ratios from all MRI sequences to identify a T2B ratio threshold algorithm for optimally predicting a true positive PICT. Predictor screening was done using bootstrap forest partition to evaluate the contribution of each characteristic on the diagnosis. Receiver operating characteristic evaluation was utilized to assess the performance of decision tree analyses through assessments of the areas under the curve.

## Results

### Reader performance

**Fig 2:**
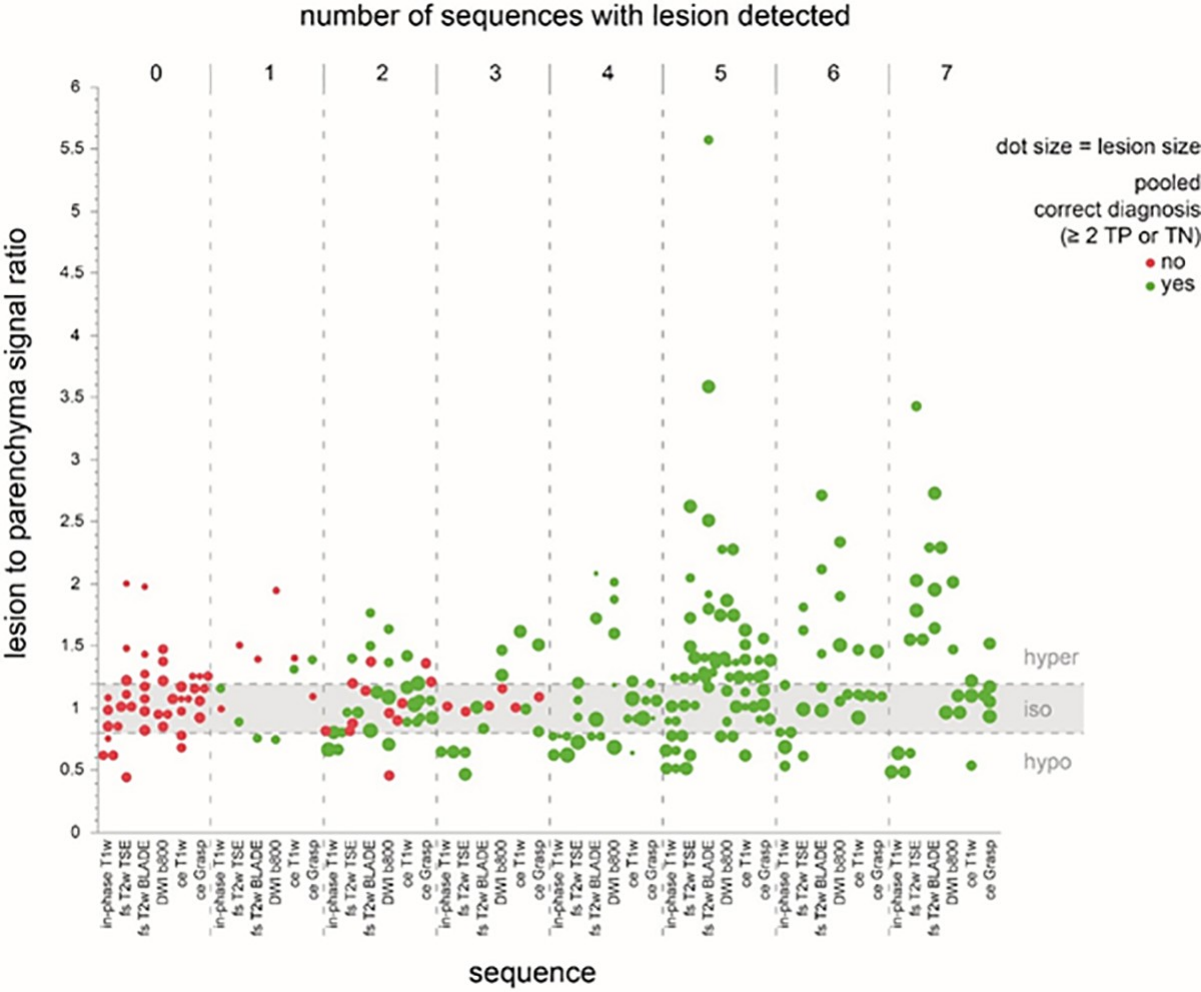
Relationship of number of sequences to correct diagnosis. The graph shows the number of sequences a lesion was detected on in relation to the measured lesion to parenchyma signal ratio. The higher the number of sequences a PICT was identified on, the more often a correct diagnosis was made. If a lesion was detected on four or more sequences, there was no missed lesion. This indicates increasing confidence in declaring a lesion to be present if detected on a larger number of sequences. Furthermore, the majority of missed lesions resides in the “iso” signal ratio corridor indicating poorly visible lesions. Correct diagnosis is indicated as green dot, wrong diagnosis as red dot. Dot size correlates with PICT size. Graph shows PICTs with obtainable measurements on imaging, therefore false positive lesions are not represented. T2w Haste coronal and axial as well as coronal ce T1w Dixon VIBE sequence are not depicted in this figure.

The pooled lesion-based accuracy, sensitivity and specificity for the three readers evaluating all 49 study MRIs were 70.3%, 72.9% and 62.5%, respectively. The pooled false positive and negative rate was 37.5% and 27.1%, respectively (Table 3). Inter-reader agreement shows substantial agreement between the three readers (Reader 1 and Reader 2: Kappa = 0.62, Reader 2 and Reader 3: Kappa = 0.73; Reader 1 and Reader 3 kappa = 0.64). Most of the total of 49 PICTs were detected on unenhanced in-phase T1w imaging (n = 33, 67.3%), followed by DWI (n = 28, 57.1%), T2w imaging (n = 28, 57.1%), ceGRASP (n = 24, 49%) and ceT1w Dixon VIBE (n = 18, 36.7%). The detection rate for PICTs identified on four or more sequences was 93.1% with 27 out of 29 PICTs correctly identified and 2 false positive readings. If PICTs were present on three or more sequences, the detection rate was 85.7%, with 30 out of 35 correctly identified PICTs and 5 false positive readings. Detected PICTs were on average conspicuous on 3.6 sequences vs. 1.3 sequences for missed PICTs, p-value 0.001 (Fig 2).

**Table 3:** Diagnostic performance for PICT detection of the three readers.

	Reader 1	Reader 2	Reader 3	Pooled
**Accuracy (%)**	64.1	66.7	74.6	70.3
**Sensitivity (%)**	66.7	72.3	77.1	72.9
**Specificity (%)**	56.3	50.0	66.7	62.5
**False positive rate (%)**	43.8	50.0	33.3	37.5
**False negative rate (%)**	33.3	27.7	22.9	27.1

Image performance is given for each reader and as the pooled lesion based results from all readers combined (majority decision).

### PICT characteristics

#### Lesion size

**Fig 3:**
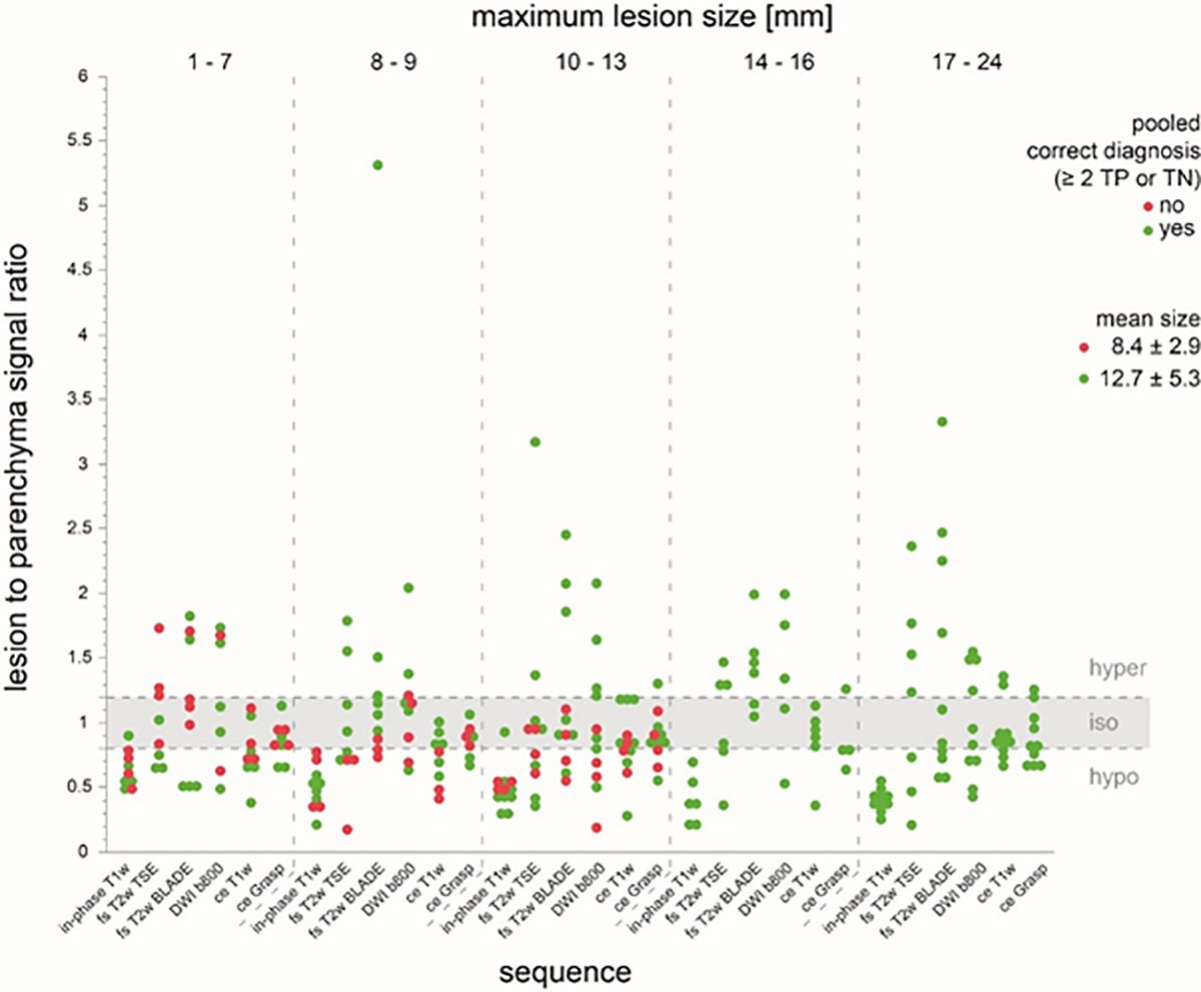
Relationship of PICT size to correct diagnosis. Correct diagnosis is indicated as green dot, wrong diagnosis as red dot. If the size is below 13mm signal characteristics are more important for the correct diagnosis. In PICTs below 13mm, fewer correct diagnoses were made in the isotime corridor. T2w Haste coronal and axial as well as coronal ce T1w Dixon VIBE sequence are not depicted in this figure.

The mean size for all PICT was 11.7 ± 5.2 mm. The PICT size differed significantly between detected PICTs with 12.9 ± 5.3mm and undetected PICTs with 9.0 ± 2.9mm (p-value 0.01) (Table 4). Above the size threshold of >13 mm all PICTs were identified by at least 2 out 3 readers (Fig 3). Below the size threshold of 13 mm, a false-negative reading occurred in 44% (12/27).

**Table 4:** Characteristics in 49 PICT compatible lesions.

Characteristics	N	Detected Lesions	Overall	Detected	Not detected	p-value
**Size** (mm) mean ± SD						
			11.7 ± 5.21	12.9 ± 5.33	9.0 ± 2.9mm	0.01*
**T2B ratio** mean ± SD						
fs T2w TSE	41	19	1.29 ± 0.58	1.34 ± 0.63	1.16 ± 0.40	0.5
in-phase T1w Dixon VIBE	47	33	0.74 ± 0.17	0.71 ± 0.17	0.82 ± 0.14	0.03*
opposed-phase T1w Dixon VIBE	47	19	0.85 ± 0.32	0.85 ± 0.35	0.83 ± 0.22	0.8
fat-only T1w Dixon VIBE	47	19	1.09 ± 0.72	1.14 ± 0.79	0.95 ± 0.46	0.5
water only T1w Dixon VIBE	47	23	0.82 ± 0.39	0.81 ± 0.44	0.84 ± 0.17	0.2
DWI b-value 50 s/mm^2^	42	28	1.42 ± 0.70	1.47 ± 0.73	1.28 ± 0.64	0.5
DWI b-value 800 s/mm^2^	42	28	1.35 ± 0.48	1.42 ± 0.49	1.12 ± 0.40	0.1
ADC	42	28	1.01 ± 0.30	0.99 ± 0.33	1.09 ± 0.19	0.2
fs T2w BLADE	45	22	1.59 ± 0.87	1.70 ± 0.96	1.23 ± 0.31	0.1
ce GRASP	41	24	1.13 ± 0.18	1.13 ± 0.21	1.14 ± 0.11	0.3
ce T1w Dixon VIBE	46	18	1.08 ± 0.24	1.11 ± 0.24	1.00 ± 0.19	0.1
**Localization**						
head			12/49 (24%)	7/35 (20%)	5/14 (36%)	
uncinate process			2/49 (4%)	2/35 (6%)	0/14 (0%)	
transition head/body			2/49 (4%)	1/35 (3%)	1/14 (7%)	
body			7/49 (14%)	3/35 (9%)	4/14 (29%)	
tail			26/49 (53%)	22/35 (63%)	4/14 (29%)	

T2B ratio = Tumor-to-background ratio, T2B ratios are only calculated for conspicuous lesions in the respective sequence. ADC = Apparent Diffusion Coefficient.

#### Tumor-to-background ratio (T2B)

**Fig 4:**
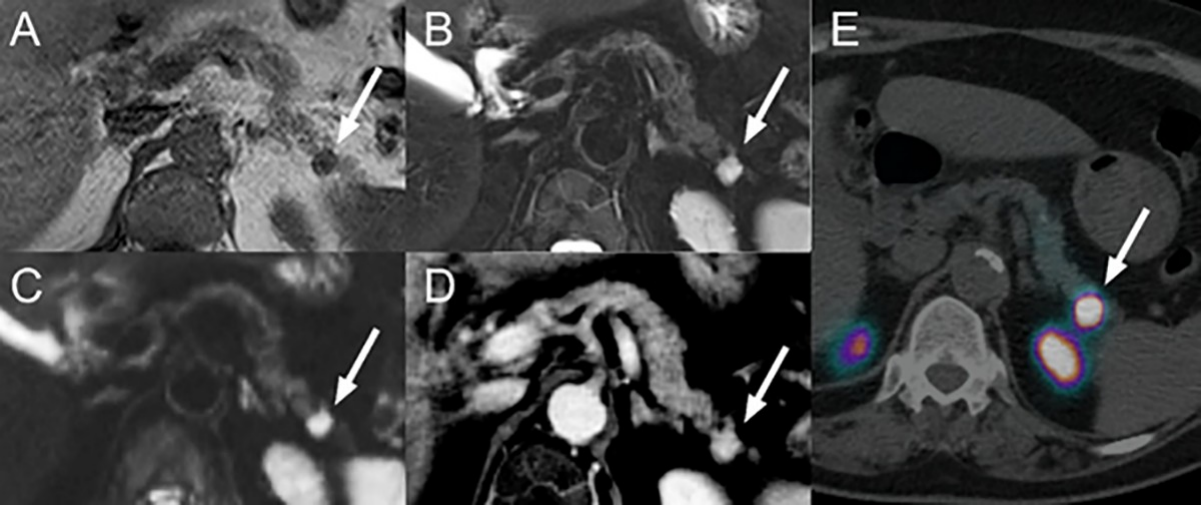
Imaging example of a small PICT with typical signal characteristics. PICT localized in the pancreatic tail (white arrow): hypointense on in-phase T1w (A), hyperintense on T2w fs T2w BLADE (B), hyperintense on DWI b-value 800 s/mm^2^ (C) and hyperenhancing on ce T1w Dixon VIBE (D). ^68^Ga-DOTA-exendin-4 PET/CT (E) confirming the lesion.

**Fig 5:**
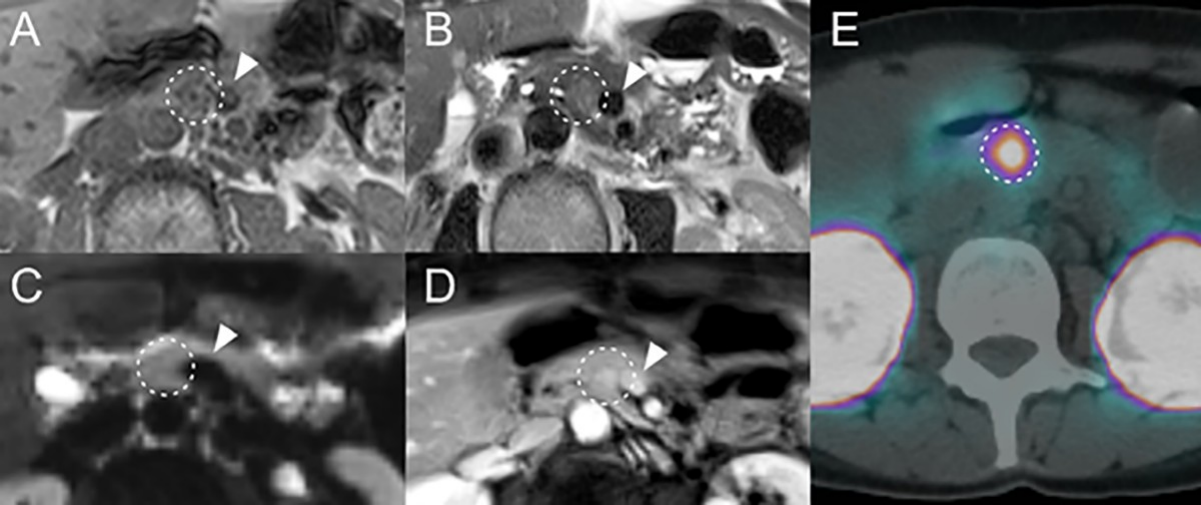
Imaging example of a small PICT with atypical signal characteristics. PICT localized in the pancreatic body (white dashed circle): isointense on in-phase T1w (A), slightly hyperintense to isointense on T2w HASTE (B), isointense on DWI b-value 800 s/mm^2^ (C) and faintly hyperenhancing to isoenhancing on ceGRASP (D). ^68^Ga-DOTA-exendin-4 PET/CT (E) confirming the lesion. The white arrowhead marks the superior mesenteric vein as reference.

A typical lesion signal behavior (T1w = hypointense and any T2w sequence = hyperintense combined) was found in 53.5% (23/43) of lesions, 46.5% (20/43) of lesions have an atypical behavior. A typical signal behavior for T1w (hypointense), T2w (hyperintense) and DWI (b-value 800 s/mm^2^; hyperintense) combined was only present in 26.2% (11/42) of lesions compared to atypical behavior in 73.8% (31/42). Combining expected typical signal characteristics for T1w (hypointense), T2w (hyperintense), DWI (b-value 800 s/mm^2^; hyperintense) and ceT1w (ce GRASP or ceT1w Dixon VIBE; hyperintense) revealed only 4.9% (2/41) of lesions compared to 95% (39/41) of lesions without typical imaging presentation across all sequences (Figs 4 and 5).

**Fig 6:**
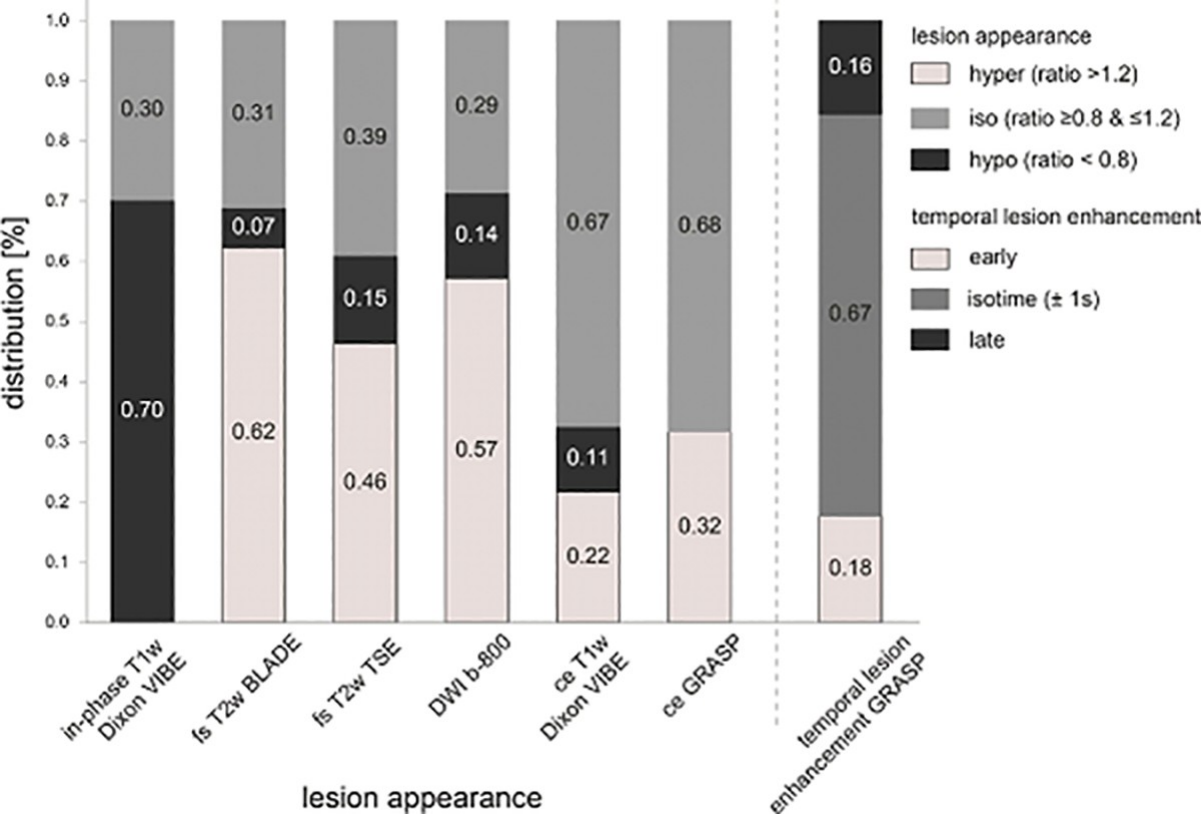
Distribution of signal characteristics of PICTs. This figure shows the distribution of signal characteristics in T1w in-phase, Fs T2w BLADE, fs T2w TSE, DWI b-800, ceT1w Dixon VIBE, ce GRASP and temporal lesion enhancement GRASP based on Tumor-to-background ratio (T2B) T2B ratios for each sequence. PICTs with a T2B ratio of < 0.8 = hypointense relative, T2B ratio of > 1.2 = hyperintense, T2B ratio ranging from 0.8–1.2 = isointense relative to normal parenchyma. The bar chart on the left depicts temporal PICT enhancement characteristics on ce GRASP sequence. Temporal enhancement was defined as early < -1 seconds, isotime between -1 and + 1 seconds and late as > +1 seconds comparing the time of maximum enhancement of the PICT to the normal pancreas.

T2B ratios for each sequence for all PICTs, detected PICTs and undetected PICTs are shown in Table 4. Per sequence typical signal characteristics of PICTs ranged from 22% to 70% with the in-phase T1w sequence showing the highest rate of typical hypointense signal lesion behavior in 70% (33/47). The lowest rate of expected typical signal behavior for non-contrast sequences was found for fs T2w TSE with 46% (19/41) of lesions demonstrating hyperintense signal. Contrast-enhanced sequences ceT1w Dixon VIBE and ce GRASP both showed a low proportion of typical hyperintense (hypervascular) lesions with 22% (10/46) and 32% (13/41) respectively (Fig 6).

**Fig 7:**
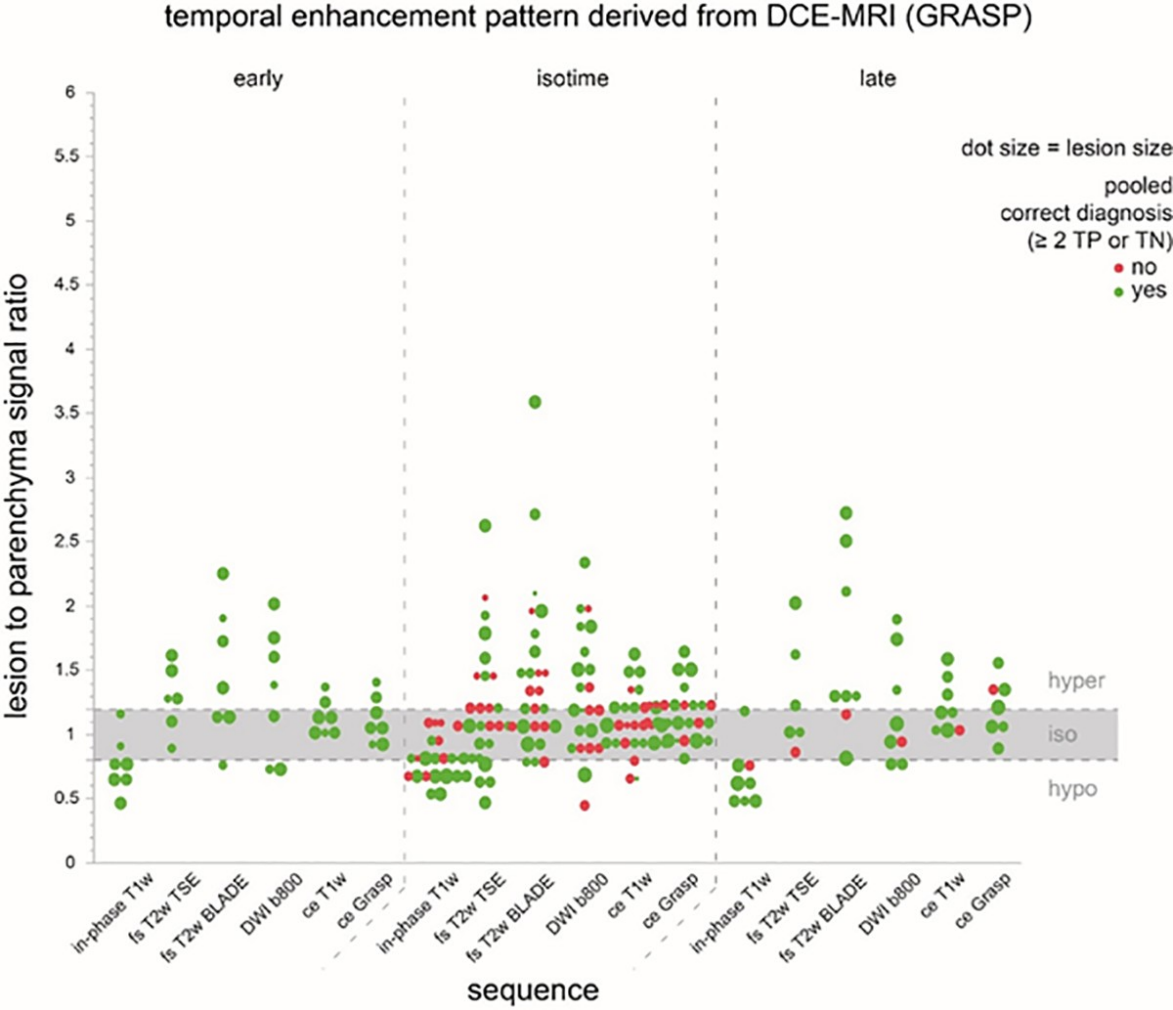
Temporal enhancement pattern of PICTs. The figures shows the temporal enhancement pattern of PICTs. Correct diagnosis is indicated as green dot, wrong diagnosis as red dot. Dot size correlates to PICT size. Temporal enhancement pattern is very variable, not all PICTs are early or late enhancing, most are isotime to the surrounding parenchyma. T2w Haste coronal and axial as well as coronal ce T1w Dixon VIBE sequence are not depicted in this figure.

Depending on the sequence, a considerable proportion of lesions were isointense with regard to the T2B ratio ranging from 29% (DWI b-800 s/mm^2^) to 68% (ce GRASP, Fig 2). For all PICTs, the sequences with the most conspicuous mean T2B ratios were unenhanced in-phase T1w (0.74 ± 0.17, hypointense), fs T2 BLADE (1.59± 0.87; hyperintense) followed by DWI (b-value 50 s/mm^2^; 1.42 ± 0.70; hyperintense). The mean T2B ratio on in-phase T1w was significantly closer to the poorly conspicuous isointense corridor (0.8–1.2) for undetected PICTs with 0.82 ± 0.14 compared to detected PICTs with 0.71 ± 0.17 (p-value 0.03). Although mean T2B ratios for T2w and DWI sequences were distinctly higher for detected versus undetected PICTs (Table 4), the difference was not significant. PICTs with a T2B ratio in the poorly conspicuous corridor (0.8–1.2; isointense) on any sequence were harder to detect when they were smaller in size (≤ 13 mm) since all larger PICTs (>13 mm) with T2B ratios in this corridor were detected (Fig 3). The majority of the PICTs are enhancing at the same time as the parenchyma (66.6%), only a minority enhances early (17.8%) or late (15.6%) in relation to the parenchyma (Fig 7).

### Predictor screening and decision tree analysis

**Fig 8:**
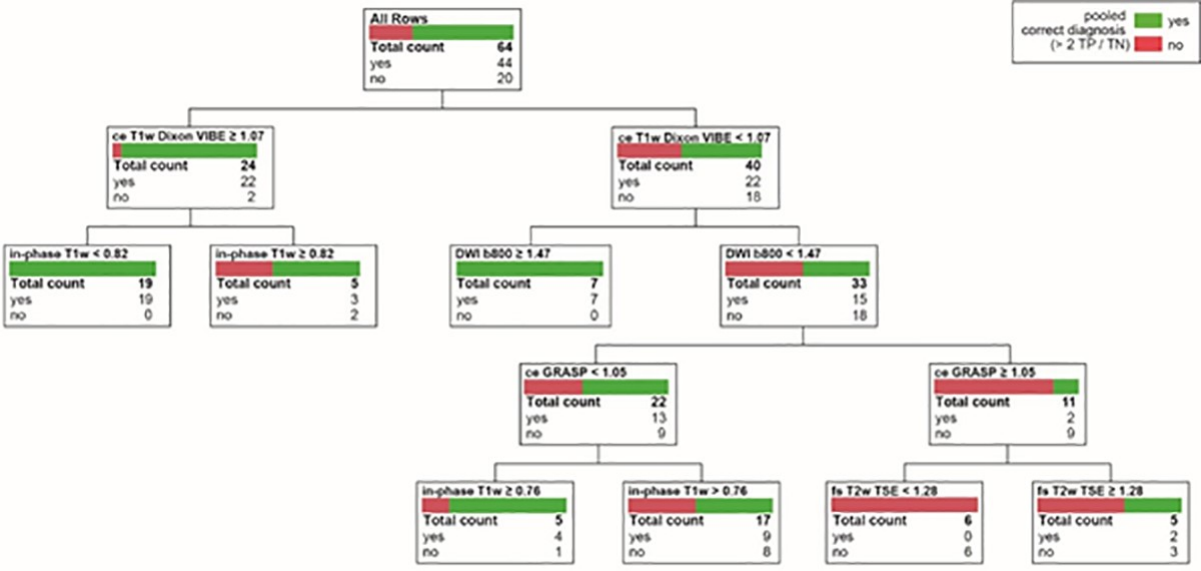
Decision tree analysis. This figure shows the decision tree analysis based on the tumor-to-background ratio thresholds of the employed sequences.

**Fig 9:**
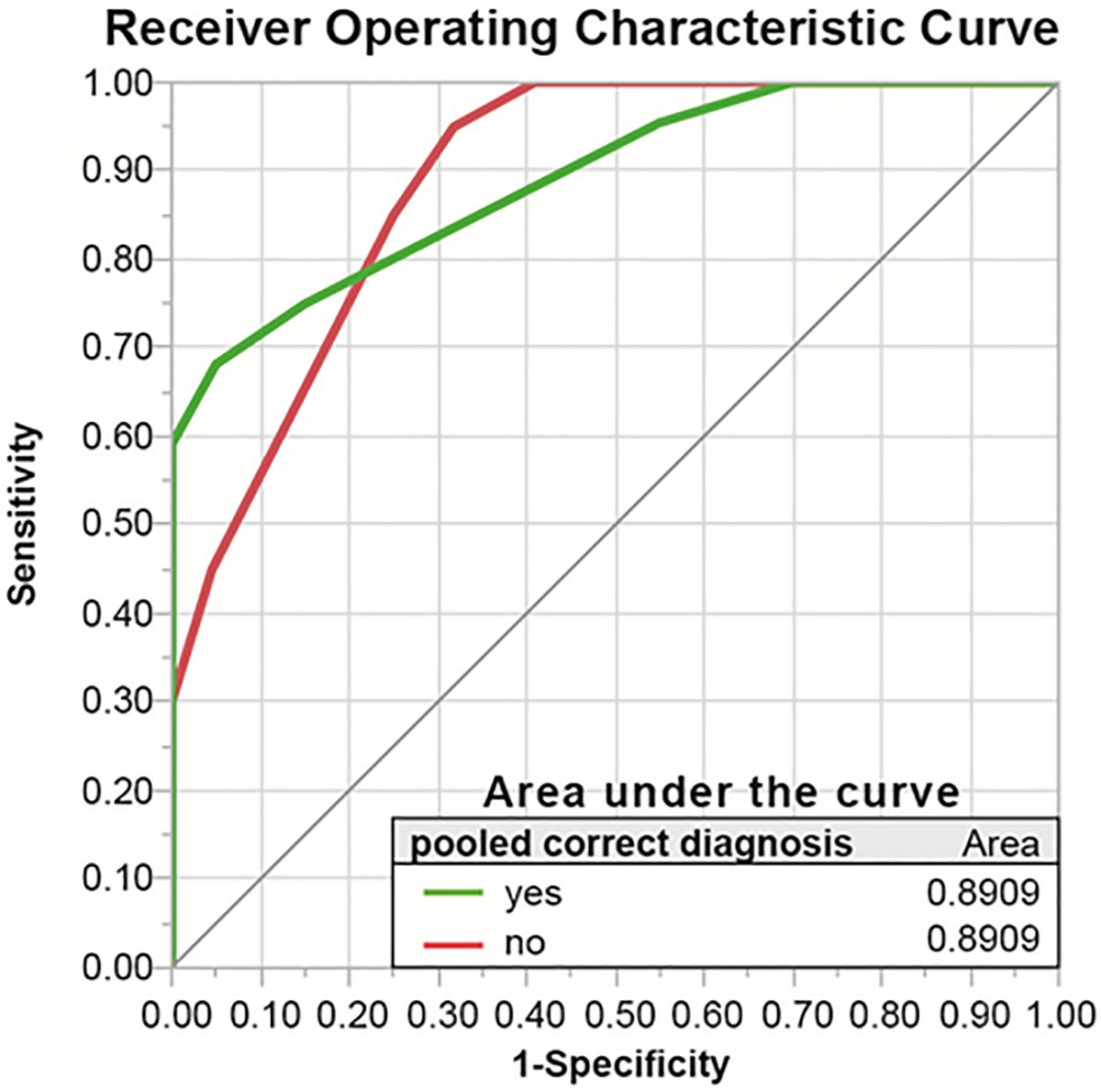
Receiver operating characteristic curve. This figure shows the receiver operating characteristic curve of the decision tree analysis based on the tumor-to-background ratio thresholds of the employed sequences.

The predictor screening showed that the number of sequences on which a PICT was detected had the largest contribution to the model (1^st^ rank, contribution 22%), followed by PICT size (contribution 17.7%), the in-phase T1w T2B ratio (contribution 11.8%) and fs T2w BLADE T2B ratio (contribution 11.4%). In a decision tree analysis, which aimed at predicting a true positive PICT based on T2B ratios, DWI, ceT1w and in-phase T1w had the highest effect on the resulting decision tree (Total effect 0.568; 0.562; 0.289 respectively). The corresponding decision tree T2B ratio thresholds for DWI, ce T1w and in-phase T1w were ≥1.47, ≥1.07, ≤0.82, respectively, supporting the a-priori chosen T2B ratio threshold definition for isointensity from 0.8–1.2 (Fig 8). The receiver operating characteristics curve for this model showed an area under the curve of 0.89 (Fig 9).

## Discussion

Our study aimed at characterizing difficult to detect PICTs (mainly insulinomas) on multiparametric 3T MR imaging and investigating factors that impede PICT detection. Our results show that in a challenging patient cohort, characterized by long-standing patients’ disease histories and prior mostly negative imaging results, a dedicated pancreatic 3T MRI which employs dynamic contrast-enhanced Magnetic Resonance Images (DCE-MRI) with Golden-Angle-Radial-Sparse-Parallel (GRASP) reconstruction can identify the majority of PICTs. However, a considerable portion of PICTs presented with atypical signal- and enhancement characteristics with regard to the expected imaging behavior.

Our study recruited patients from Europe and the United States, with most patients having prior negative imaging results, therefore focusing on difficult to detect PICTs. While larger and more typical behaving lesions are probably less challenging to detect, our results show that certain imaging features especially size, number of sequences detecting a lesion and knowledge about the variety of atypical signal behavior may help in improving detection rate for difficult to detect lesions. In case of a suspicion for a PICT based on clinical findings of endogenous hyperinsulinemic hypoglycemia (EHH) the first imaging standard of choice is typically either CT or MRI. Since ^68^Ga-DOTA-Exendin-4-PET/CT, known to have a high accuracy in insulinoma detection [10,13–15], is not widely available, it is necessary to utilize MRI’s potential to identify even small and poorly conspicuous lesions. Patients with EHH most likely have some form of pancreatic islet cell disorder either in form of a localized albeit in some instances small tumor or presenting as abnormal islet cells scattered across the pancreas, called nesidioblastosis. Since the latter is not a morphological lesion but rather caused by abnormalities on a cellular level, imaging modalities cannot detect this cause of EHH [16]. However, a small lesion in the few millimeter range may present the limit for lesion detection of current MR imaging technology [17]. This is supported by one case of this study population where a lesion was initially missed but detected on a rescan 5 years following the initial study scan.

With knowledge of the atypical lesion behavior seen in predominantly small lesions in our study population, it may be possible to improve the ability to identify difficult to detect lesions by utilizing our study results. While reading an MRI examination from a patient with EHH, radiologists should seek out the unexpected, small lesions with atypical signal behavior most likely conspicuous on unenhanced T1w or DW imaging. Our results showed that only 5% of all difficult to detect small PICT lesions demonstrate expected signal characteristics and enhancement pattern across T1w, T2w, DWI and ceT1w sequences combined.

While in this study population previously performed external CT/MRI had a low accuracy and sensitivity of 40% and 38.2%, respectively in a per-patient analysis [10], the pooled reader accuracy, sensitivity and specificity were considerably improved to 70.3%, 72.9% and 62.5% in a per lesion analysis with a substantial reader agreement (kappa Range: 0.62–0.73).

DCE T1- weighted acquisition after injection of a gadolinium-based contrast agent is an integral part in identifying and characterizing PICTs and hypervascular enhancement in the arterial phase has been described as one of the typical imaging features of PICTs [18]. However, the degree, uniformity, and timing of enhancement of PICTs can be highly variable and PICTs may be visualized at only one short time-point in the contrast-enhanced phase [19,20]. Jeon et. compared analyzed 74 histologically proven PICTs and found that 51% were hypervascular and 49% non-hypervascular [19]. On the other hand, Ichikawa et. al found that the delayed enhanced T1-weighted MR imaging obtained 5–10 minutes after contrast injection had the highest relative sensitivity (74%–79%) followed by portal-venous phase (69%-73%) in the detection of PICTs [9]. The difference in the results of these two studies maybe associated with the variable time of enhancement of PICTs [21] and consequently inappropriate timing of the enhancement phase. This shows that clinical routine DCE MRI remain challenging and is prone to failure, as data has to be collected at precisely defined time points.

To our knowledge, this study employed for the first time the compressed sensing T1w Golden-Angle Radial Sparse Parallel (GRASP) sequence for the assessment of the enhancement pattern of PICTs. This motion-robust, free-breathing sequence allows continuous acquisition during contrast-administration resulting in a high spatial and temporal resolution multiphase volume dataset. This dataset depicts the entire range of unenhanced, arterial, portal venous and late phase of contrast administration [11] eliminating the possibility of missing one important enhancement phase.

The majority of the PICTs in our study were enhancing at the same time as the parenchyma (66.6%) whilst no significant difference in the enhancement intensity of the detected and undetected PICTs was observed. Knowledge that the majority of difficult to detect PICTs enhance simultaneously to normal pancreas parenchyma is crucial for clinical routine and should prompt radiologists to search for other lesion characteristics to aid detection or increase reading confidence.

The unenhanced breath-hold in-phase T1w sequence was the most reliable sequence for PICT detection (detection rate 65%). This correlates with results of other studies, which postulate that unenhanced T1-weighted sequences provide excellent contrast between the low-signal-intensity tumor versus normal pancreatic parenchyma and thus may be the best sequence to detect subtle tumors [7,8,22]. Acknowledging the role of the T1w sequence as the most important sequence for PICT detection, it was acquired twice in our study, before and after free-breathing DW and T2 imaging. The rationale was that at the beginning of an examination, patients possibly have a better breath hold compliance and might not suffer from exhaustion caused by previous breath-hold sequences. Repeating the T1w sequence after a series of long free-breathing sequences ensures sufficient recuperation. In particular, the detection of small lesions is probably improved by reducing motion artefacts, caused by breathing, bowel motion, or patient movement otherwise lesions may be obscured by image artefacts.

However, a small lesion size is a major challenge due to not only motion artifacts but because of their conspicuity in an organ with grainy parenchymal texture and abutting vasculature that may mimic lesions. Buetow et. al evaluated the size of 133 pathologically proven PICTs and found non-functioning PICTs had a mean diameter of 77 mm, whereas functioning insulinomas had a mean diameter of 22 mm [21]. The only other prospective study we could identify with a similarly large number of PICTs compared to our study from Zhu et.al [6] reported a mean tumor size of 14 ± 6 mm in 51 evaluated PICTS. The overall PICT size in our collective was smaller than in all identified prospective MRI studies with a mean tumor size of 11.7 ± 5.2 mm, which again could be explained by our study design by including patients with mostly negative or non-conclusive prior imaging [10]. Lesion size was a relevant feature influencing PICT detection, as undetected PICTs were significantly smaller than detected PICTs (12.9 ± 5.3mm versus 9.0 ± 2.9mm; p-value 0.01).

The total number of sequences, on which a PICT was conspicuous on, played a substantial role in PICT detection and so far has only been evaluated in one prospective study 20 years ago [7]. Investigating 20 patients with a mean lesion size of 14 mm, Thoeni et al. stated that no further imaging is needed if unenhanced T1w and T2w sequences demonstrate a lesion. If no lesion is shown, ce T1w imaging should be performed with an immediate or arterial phase (20–30 seconds) and a delayed phase (1 and 2 minutes). In our study, with a mean lesion size of 11.7 mm, at least 2 out of 3 readers always detected lesions larger than 13 mm. Furthermore, the more sequences identified a PICT, the higher the detection rate and the lower the probability of a missed lesion. Translating this into clinical practice, the confidence for detecting a lesion, especially a small lesion, should increase with the number of sequences detecting a lesion.

The majority of PICTs showed a typical hypointense signal on in-phase T1w (70%), but this does not imply that typical signal characteristics are present on other sequences. Most PICTs were detected on unenhanced T1w imaging (65%), probably due to its most favorable T2B ratio among all sequences, related to the relatively high hydrogen concentration of the normal pancreatic parenchyma [7]. The second-best sequence for PICT detection was the DWI (55%) further improving tumor detection as shown in other studies [6,23,24]. In the study of Zhu et al. the sensitivity of insulinoma detection could be improved by adding DWI (sensitivity MRI without DWI 82.4% versus MRI with DWI 90.2%) [6].

Statistical analysis using a predictor screening confirmed our observed results that the number of sequences detecting a PICT, in-phase T1w imaging, as well as DWI b800-imaging, proved to have the highest contribution for PICT detection. Therefore, a MRI protocol should be sufficiently robust and allow for detection of small lesions by employing the full spectrum of diagnostic imaging including T1w, T2w, DWI and ce T1w sequences as well as repeat scans and high temporal resolution multiphase post contrast imaging if available.

### Limitations

There are some noteworthy limitations of this study. Due to the design of this study, mainly difficult cases with previous negative or non-conclusive CT or MRI imaging results were included, which could have resulted in a negative selection bias and may increase the number of cases without histopathological confirmation. Therefore, the proportion of clearly conspicuous and larger insulinomas may be low in this study compared to other studies. Lesion characteristics in these tumors might be different, possibly influencing the results of our study. However, other studies have shown similar results with a proportion of lesions presenting with atypical imaging characteristics [8,9]. Nevertheless, patients with biochemically proven EHH most likely have some form of dysfunction in pancreatic islets cells either as localized tumor, which may be too small to detect, or in form of hypertrophic beta-cells cells called nesidioblastosis. In this sense, our study results do not reflect a diagnostic performance based on an available ground truth but should be evaluated in contrast to the employed reference standard, ^68^Ga-DOTA-exendin-4 PET/CT with its own limitations regarding accuracy, sensitivity and specificity. ^68^Ga-DOTA-Exenind-4 PET/CT is not a routine imaging procedure in clinical practice; however, using it as a reference standard can be justified as it had a substantial higher accuracy than 3T MRI in the preoperative localization of insulinomas [10]. Reading was performed in a study setting instead of a clinical setting in which readers were aware that MRI was performed in search for an insulinoma. False-positive reading in this study setting might be higher compared to other studies, in a clinical setting more false-negative readings may be expected. Also only benign insulinomas were evaluated in this patient collective and imaging features in malignant insulinomas may differ.

### Conclusion

In this patient collective with difficult to detect PICTs and mostly negative external prior imaging only a minority of lesions showed expected imaging behavior across T1w, T2w, DWI and ceT1w sequences combined. The majority of PICTs enhance simultaneously to surrounding parenchyma, present with atypical signal-characteristics with regard to expected imaging behavior rendering them difficult to detect. In case of clinical suspicion for an EHH, radiologist should search for small lesions most likely conspicuous on unenhanced T1w or DW imaging.

DCE-MRI using GRASP reconstruction reduces the possibility of missing the time-point of the enhancement phase and should be applied in imaging of PICTs. In non-enhancing PICTs, radiologists should search for small lesions most likely conspicuous on unenhanced T1w or DWI. Knowledge about this imaging behavior in small difficult to detect lesions can increase awareness and improve detection rates in the clinical routine.

## Supporting information

S1 File (XLSX)
